# Non-invasive in vivo imaging of porcine islet xenografts in a preclinical model with [^68^Ga]Ga-exendin-4

**DOI:** 10.3389/fnume.2023.1157480

**Published:** 2023-05-02

**Authors:** Felix Lindheimer, Magdalena Julia Lindner, Rosel Oos, Mohsen Honarpisheh, Yichen Zhang, Yutian Lei, Lelia Wolf-van Buerck, Franz Josef Gildehaus, Simon Lindner, Peter Bartenstein, Elisabeth Kemter, Eckhard Wolf, Jochen Seissler, Sibylle Ziegler

**Affiliations:** ^1^ Department of Nuclear Medicine, University Hospital of Munich, LMU Munich, Munich, Germany; ^2^ Medizinische Klinik und Poliklinik IV, Diabetes Center, University Hospital of Munich, LMU Munich, Munich, Germany; ^3^ Chair for Molecular Animal Breeding and Biotechnology, Gene Center and Department of Veterinary Sciences, LMU Munich, Munich, Germany; ^4^ Center for Innovative Medical Models (CiMM), LMU Munich, Oberschleissheim, Germany; ^5^ German Center for Diabetes Research (DZD), Neuherberg, Germany

**Keywords:** PET, exendin-4, xenografts, transplantation, autoradiography

## Abstract

**Introduction:**

Islet xenotransplantation may be a therapeutic option in type 1 diabetes. Recent advances in generating genetically modified source pigs offer advantages as immune suppressants can potentially be eliminated after the transplantation. Therapy monitoring would greatly benefit from noninvasive methods for assessing the viability of transplanted islets. Peptide-based positron emission tomography (PET) targeting the glucagon-like peptide-1 receptor (GLP1R) expression on beta cells may offer a procedure that can directly be translated from an experimental setting to the clinic. The aim of this study was to establish the labeling of the GLP1R ligand [^68^Ga]Ga-exendin-4, to demonstrate the feasibility of imaging porcine islet xenografts *in vivo* and to compare signal quality for three different transplantation sites in a mouse model.

**Materials and methods:**

Mice with engrafted neonatal porcine islet cell clusters (NPICCs) under the kidney capsule, into the inguinal fold, or the lower hindlimb muscle were studied. After reaching normoglycemia, the mice were injected with [^68^Ga]Ga-exendin-4 for PET data acquisition. Subsequent autoradiography (AR) was used for comparing *ex vivo* data with *in vivo* uptake.

**Results:**

NPICCs in the lower right hindlimb muscle could be detected *in vivo* and in AR. Due to the high background in the kidney and urinary bladder, islets could not be detected in the PET data at transplantation sites close to these organs, while AR showed a clear signal for the islets in the inguinal fold.

**Discussion:**

PET with [^68^Ga]Ga-exendin-4 detects islets transplanted in the hindlimb muscle tissue of mice, offering the potential of longitudinal monitoring of viable porcine islets. Other sites are not suitable for *in vivo* imaging owing to high activity accumulation of Exendin-4 in kidney and bladder.

## 1. Introduction

The number of type 1 diabetes (T1D) cases worldwide has increased significantly since the 1950s and is still increasing by more than 3% per year in the early 21st century (1, 2). T1D results from complete autoimmune destruction of insulin producing beta cells leading to absolute insulin deficiency and the need for therapy with insulin injections or continuous insulin infusion *via* insulin pumps, such as the insertion of a bio artificial pancreas (BAP), to overcome the malfunction of the pancreas (3, 4). However, despite continuous technical improvements in BAP technologies, blood sugar control in a small fraction of T1D patients with brittle diabetes is still inappropriate as unpredictable life-threatening events of hypoglycemia occur in these patients (5).

In recent years, there has been an upcoming focus on a new strategy including cell replacement therapies with isolated islets or beta cells derived from embryonic or induced pulipotents stem cells. As there is a significant lack of human islet donors, genetically modified islets from other species such as genetically modified pigs became of more interest (5, 6). Recent studies have already proven that neonatal porcine islet-like cell clusters (NPICCs) may represent a promising alternative beta cell source for islet transplantation (7, 8).

Xenograft donor pigs could be genetically modified in such a way that the graft can be tolerated by the human recipient's body and graft rejection is avoided, as recently demonstrated by the first pig-to-human heart xenotransplantation in 2022 (9). Genetic modifications of islet donor pigs are aimed at establishing a local immunoprotective microenvironment protecting free islet xenotransplants against graft rejection despite omitting a systemic immunosuppressive regime (6). Monitoring the graft fate not only by functional data such as glucose or C-peptide levels but also by non-invasive monitoring the viable beta-cell mass (BCM) and follow-up of changes in BCM over time is of high clinical relevance (10).

Beta cells express on their surface the glucagon-like peptide receptor-1 (GLP1R). GLP1R imaging was successfully performed to detect insulinomas in human patients by using radionuclide-labeled exendin-4, an analogue of the metabolically unstable endogenous ligand glucagon-like peptide-1 (GLP1) (11). Binding and internalization of radionuclide-labeled exendin-4 by GLP1R positive cells was favored when exendin-4 was labeled at the lysins either at position 12 or 40 (12). A biodistribution study of ^68^Ga-labeled exendin-4 in mice revealed high specific uptake and signal accumulation in the lung and pancreas as well as to a lower degree in the stomach and intestine, whose signal could be blocked when applying unlabeled excendin-4 shortly prior to tracer application, whereas the strong signal in the kidney as an excretion organ could not be blocked (12). This biodistribution pattern of exendin-4 GLPR imaging was in line with the reported GLP1R expression pattern (13). Insulinoma theranostics has been a focus of exendin-4 imaging, as summarized in a recent review by Jansen et al*.* (14). Pancreatic beta-cell imaging using exendin-4 has been performed using PET in animal models (15). Eter et al*.* showed the feasibility of *in vivo* determination of viable beta-cell graft in mice using single photon emission computer tomography (SPECT) with ^111^In-labeled exendin-3 (16). Furthermore, a transplant of human islet cells in the liver of diabetic mice using [^68^Ga]Ga-DO3A-VS-Cys^40^-exendin-4 for PET imaging has been performed and has shown the general suitability to treat hyperglycemia with transplanted islet cells and to use exendin-4 as tool of choice during the monitoring process (17).

This study aimed to investigate the feasibility of *in vivo* beta-cell imaging of pig islet xenografts at different transplantation sites in a NOD-SCID IL2r*γ*^−/−^ (NSG) mouse model using PET/CT. As a peptide for binding on porcine GLP1R, we chose ^68^Ga-labeled [Nle^14^,Lys^40^(Ahx-DOTA)NH_2_]exendin-4 with high molar activity to improve the radiosignal in the graft (18). ^68^Ga is readily available *via* a ^68^Ge/^68^Ga-generator and is routinely applied in clinical and preclinical PET imaging. Autoradiography (AR) was used as *ex vivo* reference to the PET signals.

## 2. Materials and methods

### 2.1. Peptide and chemicals

[Nle^14^,Lys^40^(Ahx-DOTA)NH_2_]exendin-4 (exendin-4) was chosen as it shows good binding affinity and high specific uptake in GLP1R-positive tissue (18), and the peptide showed high specific binding on porcine GLP1R (19). The peptide was synthesized by Peptide Speciality Laboratories (Heidelberg, Germany). Ultrapure water and absolute Ethanol (gradient grade for HPLC) were purchased from Merck (Darmstadt, Germany). HEPES was obtained from AppliChem (Darmstadt, Deutschland). Phosphate-buffered saline (PBS) was purchased from B. Braun SE (Melsungen, Germany). [^68^Ga]GaCl_3_ was eluted from a ^68^Ge/^68^Ga-Generator with 0.1 M hydrochloric acid from Eckert & Ziegler Radiopharma (Berlin, Germany).

### 2.2. Radiolabeling

350 µl [^68^Ga]GaCl_3_ eluate (190.0 ± 13.2 MBq) from the main fraction between 1.5 ml and 2.5 ml of the total elution (needleless eluted) were added to a 1.5 ml Eppendorf tube containing 100 µl HEPES-buffer (2.5 M, pH 7) and 2 µl exendin-4 (0.2 mM in ultrapure water, 0.4 nmol). The solution was mixed for 10 min at 95°C and 900 rpm and the radiochemical yield determined *via* HPLC (Agilent Series 1,200, Chromolith RP-18e, 100 mm×4.6 mm, flow rate 2.0 ml/min, 100% H_2_O + 0.1% TFA to 100% MeCN + 0.1% TFA within 7 min). Purification was performed with a 50 mg C8 SPE cartridge from Waters (Boston, USA). 2 ml ultrapure water were used to equilibrate the cartridge. After loading, the cartridge was eluted with 2 ml water and fractionally with 600 µl EtOH/H_2_O 1:4, 75 µl EtOH/H_2_O 1:1, and finally 300 µl EtOH/H_2_O 1:1, which contains the main activity with more than 99.9% radiochemical purity (checked *via* HPLC). The last fraction was formulated with PBS to receive less than 10% EtOH in the final product.

### 2.3. Animal experiments

#### 2.3.1. Animals

Experiments were performed in streptozotocin-diabetic (180 mg/kg body weight, one-time intraperitoneal injection) immunodeficient NOD-SCID IL2r*γ*^−/−^ (NSG) mice. Mice were purchased from The Jackson Laboratory (Bar Harbor, Maine, USA) and housed two animals per cage with free access to food and water. The housing room was temperature- and humidity-controlled with a 12 h cycle of night and daytime.

#### 2.3.2. Transplantation

NPICCs from 1 to 5 days-old piglets were isolated and *in vitro* cultured as described before (8, 20). 2,500–3,000 NPICCs were transplanted into three different transplantation sites, the left kidney (subcapsular), right inguinal fold (subcutaneous space), or the right hindlimb muscle (intramuscular). These sites are typical locations for islet grafts in ongoing experiments in diabetic mice. All experiments were approved by the local animal care committee and were performed in agreement with Directive 2010/63/EU.

#### 2.3.3. PET imaging

After seven to ten weeks of *in vivo* maturation, when a normal intraperitoneal glucose tolerance test was achieved, the mice were transferred to the department for nuclear medicine of the hospital of the LMU University. After an adjustment period of two weeks, the mice were anesthetized with 2% isoflurane and placed on a small animal PET/CT scanner (nanoscan PET/CT, Mediso Ltd., Hungary). During the imaging process, the narcosis was maintained with 1.5% isoflurane in a flow of 2 L/min oxygen. The radioactivity was injected through the lateral tail vein and the mice were scanned for 40 min. CT scans covering the whole animal were acquired for anatomical localization and attenuation correction of the PET data. PET data were reconstructed in 10 × 1 min, 10 × 2 min and 1 × 10 min frames, including detector normalization, corrections for random and scattered coincidences, as well as attenuation.

Owing to non-specific uptake in tissue close to the transplantation site, graft detection and beta cell mass quantification may be affected by surrounding tissue and relative total activity in the graft volume. Furthermore, the total injected peptide dose determines the proportion of blocked receptors and is defined by the injected activity with molar activities reached in the labeling process. To cover a wide range of imaging situations, injected activities ranged between 1.4 and 37.5 MBq: 1) in mice with graft in the hindlimb muscle (*n* = 3) between 1.4 and 2.3 MBq with molar activities between 232.9 and 340.6 MBq/nmol which corresponds to peptide-to-subject ratios in the range of 0.9 to 1.2 µg/kg body weight, 2) in mice with subcapsular kidney graft (*n* = 3) on average 24.7 MBq with 318.8 MBq/nmol and 13.6 µg/kg, and 3) the mouse with the inguinal fold graft (*n* = 1) 22.8 MBq with a molar activity of 360.0 MBq/nmol and a peptide dose of 10.9 µg/kg were applied. All peptide doses were well below the competing dose as defined by Eriksson et al*.* for mice (21).

PET images were analyzed quantitatively using Standardized Uptake Values (SUVs), considering the activity concentration in the PET image, injected activity, and animal weight (PMOD Technologies LLC, Zurich, Switzerland). Volumes of interest (VOI) were defined manually on fused PET/CT images at the locations of the islet grafts (spherical, *r* = 2 mm) and in organs without engrafted NPICCs (liver, kidney, leg muscle, inguinal fold; *r* = 2 mm), guided by the anatomical information on the CT images. For the leg grafts, VOIs were drawn manually to better fit the transplantation site and reduce background signal. In addition, reference volumes were defined in contralateral location. Mean values in the regions were compared for the three transplantation sites. The ratio of mean concentration in the graft vs. reference region was determined as a function of time after injection of the tracer.

#### 2.3.4. *Ex vivo* Autoradiography (AR)

Directly after the scan, the mice received an overdose of isoflurane (5 L/min isoflurane, 2 L/min oxygen) and were euthanized *via* cervical dislocation. The organs were removed, embedded on Tissue-Tek O.T.C., and frozen on dry ice. 16 µm thick slices were cut on a Leica CM1510 Kryostat at −20°C immediately taken up with a microscope slide, placed on a photo plate and analyzed after 24 h with a CR-Reader (v.1.4.1, Elysia-raytest GmbH) and an Aida Image Analyzer software (v.4.50.010, Elysia-raytest GmbH).

## 3. Results

### 3.1. Radiolabeling

The radiochemical yield was in a range between 51% and 98% (av. 86.4%). Purification gave final fractions of up to 300 MBq with more than 99% radiochemical purity with molar activities between 232.9 and 459.3 MBq/nmol (av. 312.4 MBq/nmol) at the time of injection.

### 3.2. PET imaging

**Figure 1:**
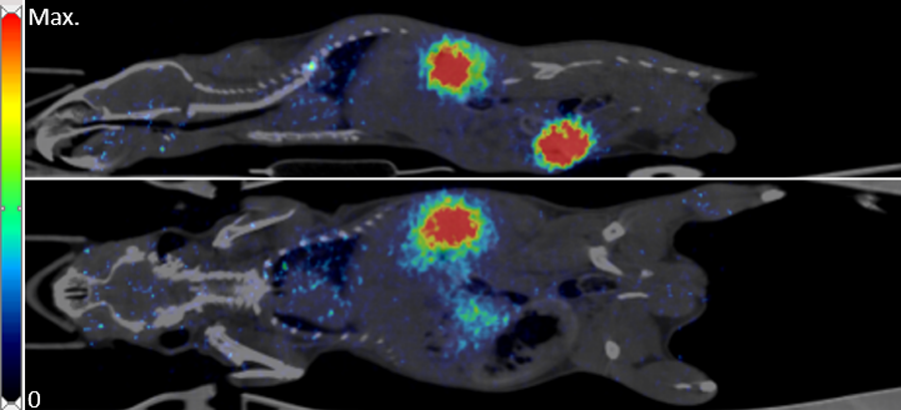
Example of [^68^Ga]Ga-exendin-4 distribution in a mouse with NPICC in the leg muscle. PET-data (30-40 min) fused with corresponding CT. Top: Sagittal slice; bottom: Coronal slice.

Figure 1 shows an example of a late (30–40 min p.i.) PET image with high activity concentration in the kidneys and urinary bladder and a weak signal in the lung and liver.

**Figure 2:**
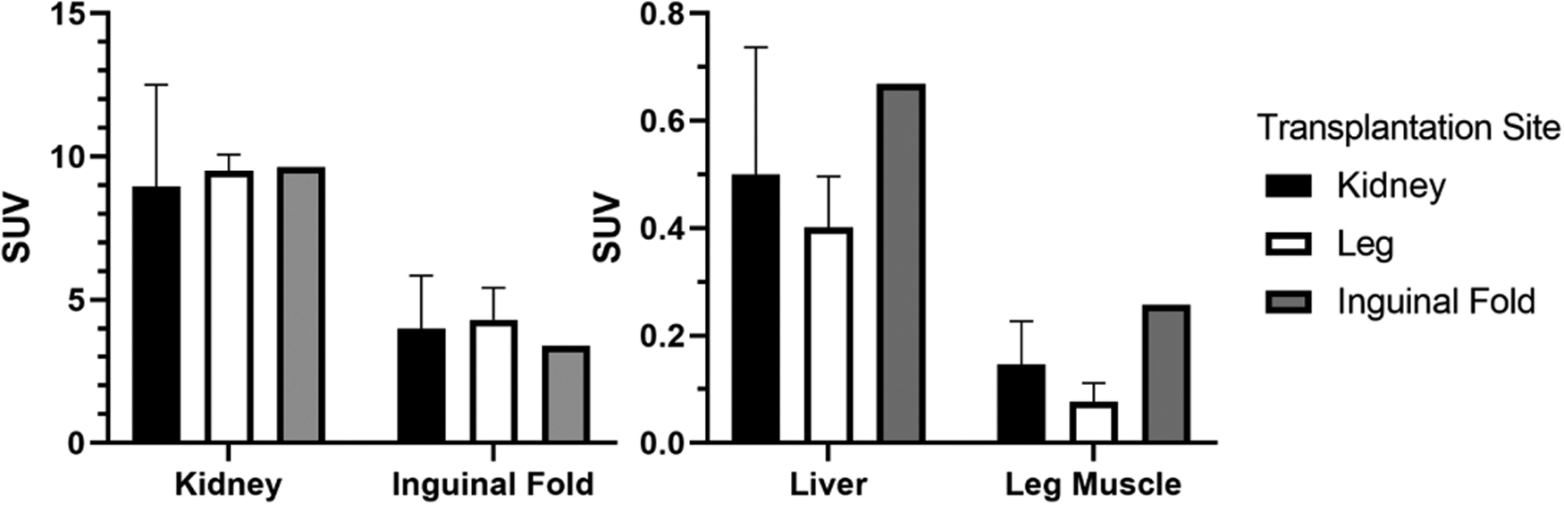
Average SUV in different investigated transplantation sites and the liver (final frame from 30 to 40 min, kidney/leg muscle: *n* = 3, inguinal fold: *n* = 1).

This is also reflected in the quantitative data. SUV in organs without engrafted NPICCs show high uptake in the kidney and the inguinal fold region but only small accumulation in the liver and leg muscle (Figure 2).

**Figure 3:**
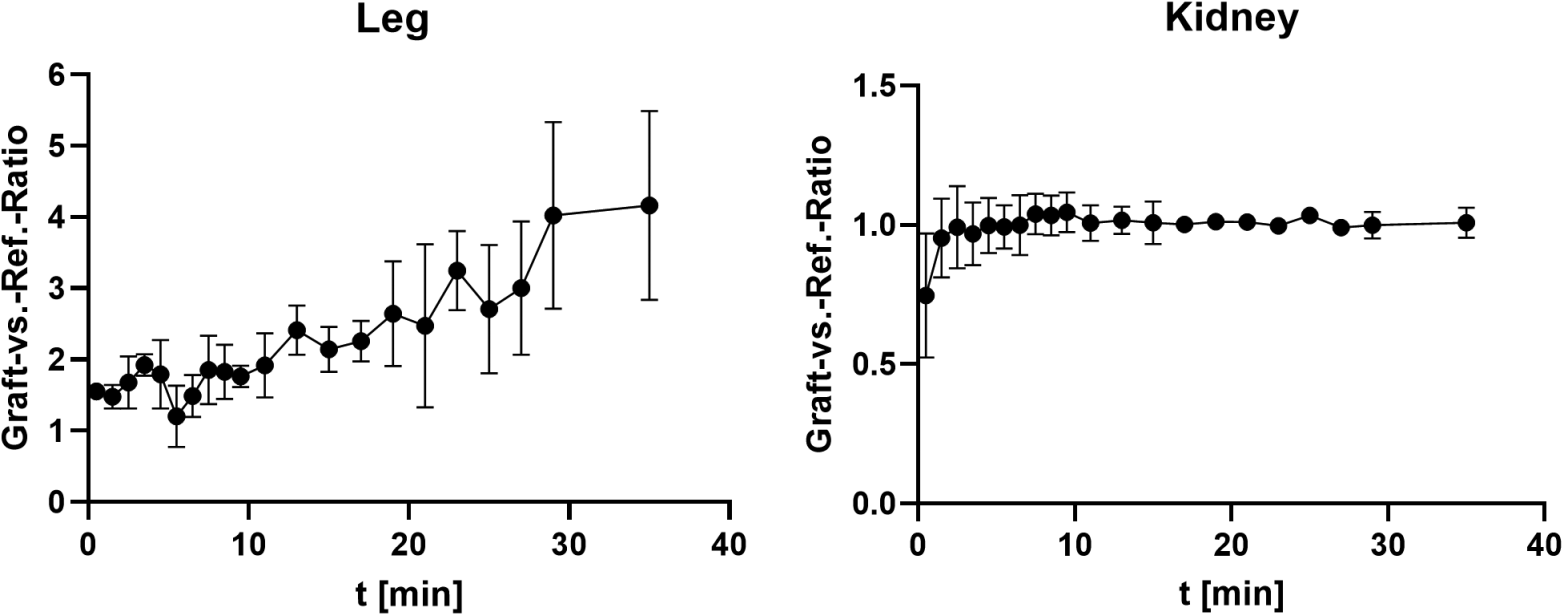
Ratio between the mean uptake in graft and reference region (*r* = 2 mm, averaged, *n* = 3).

The dynamic scan shows a continuous increase of activity concentration in ICC vs. the reference region up to 30 min in the animals with graft in the leg muscle but not the kidney (Figure 3). For the inguinal fold graft, the ratio could not be determined because of the volume increase of the bladder during the scanning process, which makes a graft localization challenging.

**Figure 4:**
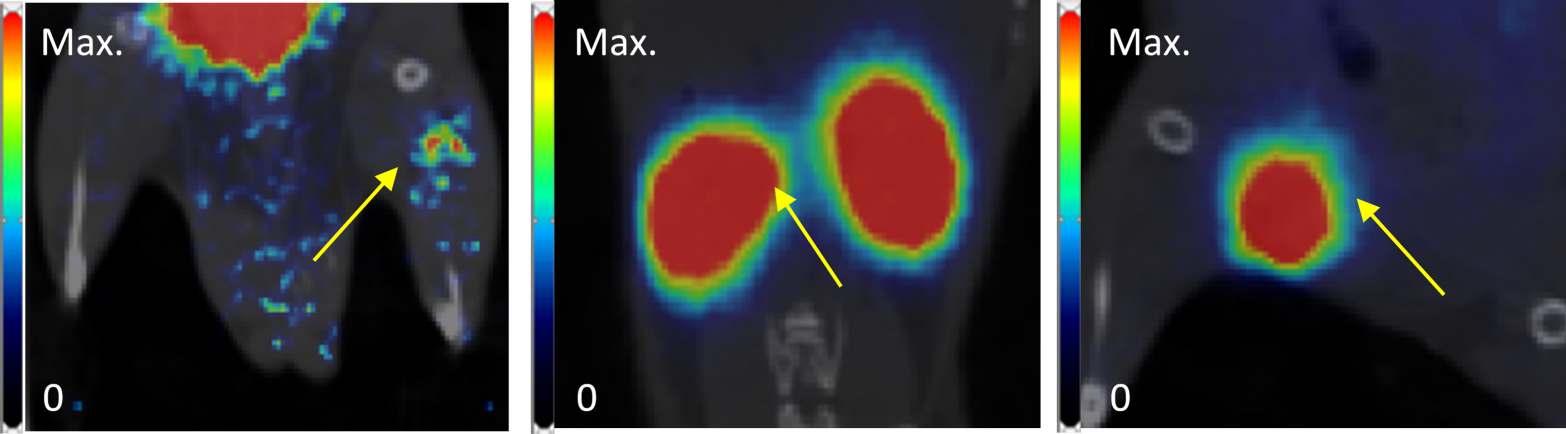
Examples of PET images (coronal slice, frame 30 to 40 min p.i., superimposed on coregistered CT images) of NPICCs in different transplantation sites (left to right: leg, kidney, inguinal fold). Yellow arrow points to the transplantation site.

Examples of late (30–40 min p.i.) PET-images are shown for all three transplantation sites in Figure 4. Only in mice where NPICCs were transplanted to the leg muscle was a signal in the region of the graft clearly detectable.

**Figure 5:**
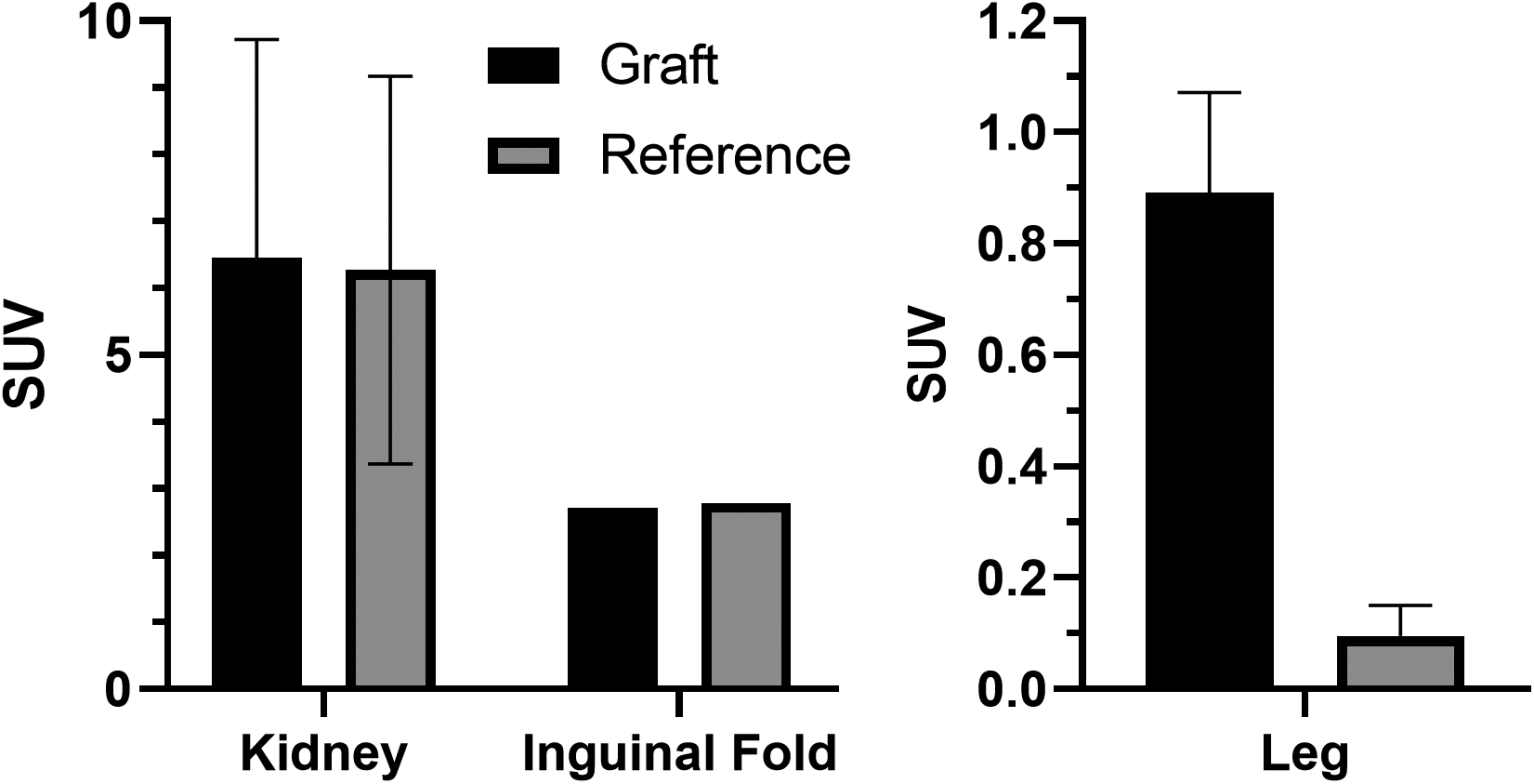
SUVs in the graft region and reference tissue after reaching a plateau (final frame from 30 to 40 min). Left: Average of mean SUV in spherical volume (*r* = 2 mm); right: Average of mean SUV in the leg grafts vs. average in the muscle (*r* = 2 mm; *p* < 0.001).

Average results of the mean SUV in the graft region at the last frame are given in Figure 5. A significant difference in SUV (*p* < 0.001) could only be found for grafts in the leg muscle using the mean uptake of a manually adapted VOI to reduce the background signal and better fit the graft region. This results in a graft-vs.-reference ratio of about 18-to-1 (Figure 5).

### 3.3. *Ex vivo* Autoradiography (AR)

**Figure 6:**
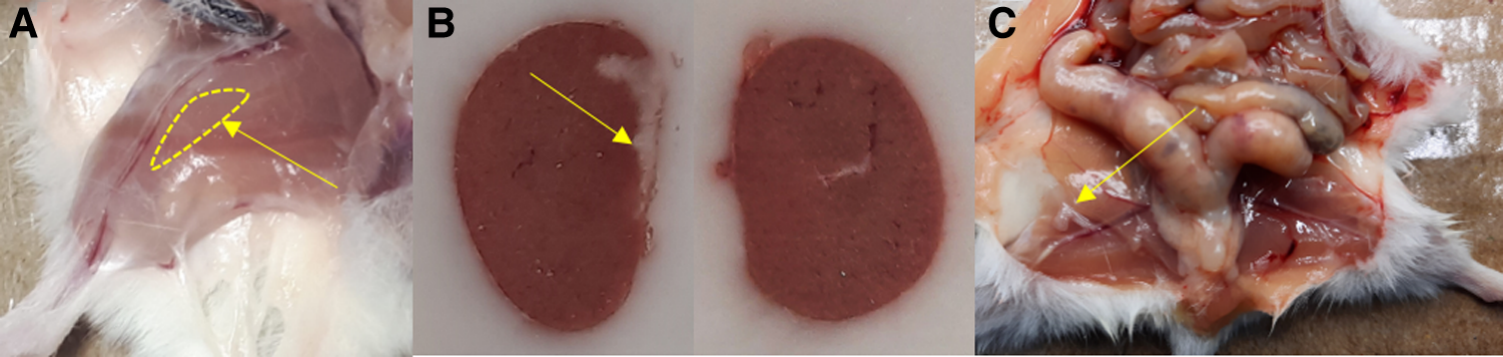
Localization of the graft in (**A**) leg muscle, (**B**) the left kidney (left; comparison with the right kidney), and (**C**) the inguinal fold during the section. Yellow arrows point to the site of transplantation, in case of the leg muscle the region where the graft was expected.

The grafts in the kidney and the inguinal fold could be clearly localized visually during the dissection (Figure 6).

**Figure 7:**
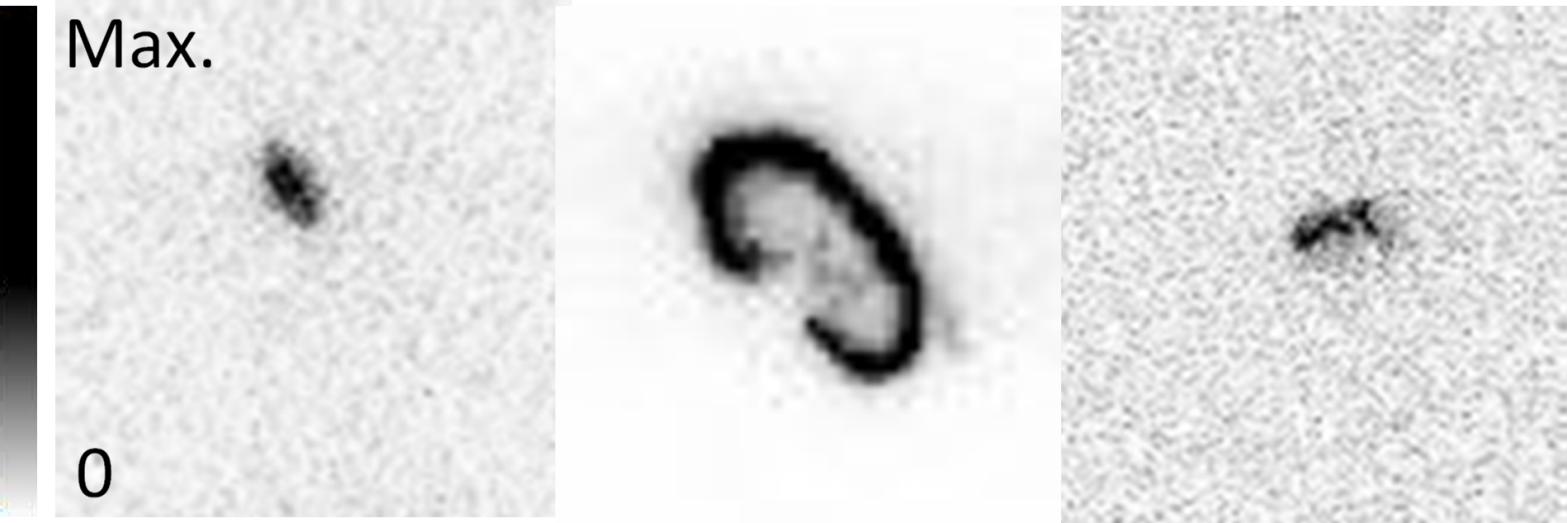
Examples of AR results in the graft regions in (left to right) leg muscle, kidney, and inguinal fold.

The NPICCs in the hindlimb muscle could not be distinguished from the surrounding tissue by eye during dissection but were clearly detectable in the AR (Figure 7). The signal of the NPICCs engrafted into the kidney could not be distinguished from the high uptake of the organ in the AR. The engrafted NPICCs in the inguinal fold were clearly detected in the AR.

## 4. Discussion

For the first time, *in vivo* imaging of porcine islet grafts by PET/CT using [^68^Ga]Ga-exendin-4 was demonstrated in this study. However, distance of graft location from tissue sites with high endogenous exendin-4 signal, either by GLP1R specific uptake or by its function as excretion organ, is of major importance. The radiolabeling of exendin-4 with Gallium-68 was successful with good radiochemical yields and high molar activities, which allowed us to work with high activities and low peptide-to-subject ratios. The purification process led to highly concentrated fractions with up to 1 MBq/µl.

Only NPICC transplants in the leg muscle could be detected *in vivo* with high uptake of [^68^Ga]Ga-exendin-4 in the mouse model. While AR proved the positive uptake in transplanted NPICCs in the inguinal fold, no *in vivo* signal could be detected because of the proximity of the urinary bladder. Background in the bladder could potentially be reduced using urinary catheterization or by implementing a protocol in which the mouse is allowed to wake up after the accumulation phase to empty the bladder and afterwards to undergo another anesthesia and PET scan. The kidney capsule was unsuitable for *in vivo* detection of viable beta cells *via* [^68^Ga]Ga-exendin-4 imaging, as there was not even an additional graft-derived separable AR signal detectable close to the extremely high uptake of [^68^Ga]Ga-exendin-4 in the kidney cortex due to the presence of GLP1R in the glomerular capillary and vascular walls of the mouse kidney (22). This makes the kidney a less suitable site for the *in vivo* imaging of NPICCs.

The high contrast of graft vs. background in the leg muscle may facilitate monitoring of beta cell viability using PET with exendin-4. In a model using adult human islets transplanted in the livers of mice, Li et al. reported a PET signal ratio of about 4 to 1 with a similar radioligand (17). Since the BCM at time of imaging is not known, a direct comparison is not meaningful. However, the high contrast measured in the region of the leg graft could indicate that maturation of NPICCs has occurred and GLP1R is expressed in the graft islets. Contrast could further be increased by a higher amount of transplanted NPICCs as a linear correlation between BCM and SUV has been reported before (23). Thus, future experiments on longitudinal *in vivo* monitoring may benefit from using the maximum value of [^68^Ga]Ga-exendin-4 as indicator and higher amounts of transplanted NPICCs.

Eter et al*.* investigated adult murine islet cell grafts in mice with an Indium-111-labeled exendin for SPECT-imaging and showed a linear relationship between graft volume and signal strength (16). In our model using NPICCs and PET-imaging, we could clearly quantify a signal in the leg, which indicates the presence of viable beta cells in this region. Future studies will focus on correlating the quantitative PET-signal with histological findings.

As the liver only shows low accumulation and is better perfused than the leg muscle, it may be a more favorable location to investigate islet cell transplantation in future studies, although intraportal access is challenging in the mouse.

In conclusion, to monitor the long-term survival and stability of transplanted NPICCs *in vivo* with radiolabeled exendin-4 a sufficient distance to the excretory organs is required if the signals of these organs cannot be suppressed and therefore the limb muscle has proven to be the most suitable transplantation site in this study.

## Data Availability

The raw data supporting the conclusions of this article will be made available by the authors, without undue reservation.
